# Visualizing the third dimension in virtual training environments for neurologically impaired persons: beneficial or disruptive?

**DOI:** 10.1186/1743-0003-9-73

**Published:** 2012-10-05

**Authors:** Wouter van den Hoogen, Peter Feys, Ilse Lamers, Karin Coninx, Sofie Notelaers, Lore Kerkhofs, Wijnand IJsselsteijn

**Affiliations:** 1 Game Experience Lab, Human Technology Interaction group, Eindhoven University of Technology, PO Box 513, Eindhoven, 5600MB, The Netherlands; 2 REVAL Rehabilitation Research Centre, PHL University College, and BIOMED Biomedical Research Institute, Hasselt University, Agoralaan building A, Diepenbeek, BE-3590, Belgium; 3 Hasselt University – tUL – IBBT, Expertise Centre for Digital Media, Wetenschapspark 2, Diepenbeek, BE-3590, Belgium; 4 Rehabilitation and MS Centre Overpelt, Boemerangstraat 2, Overpelt, 3900, Belgium

## Abstract

**Background:**

Many contemporary systems for neurorehabilitation utilize 3D virtual environments (VEs) that allow for training patients’ hand or arm movements. In the current paper we comparatively test the effectiveness of two characteristics of VEs in rehabilitation training when utilizing a 3D haptic interaction device: Stereo Visualization (monoscopic vs stereoscopic image presentation) and Graphic Environment (2.5D vs 3D).

**Method:**

An experimental study was conducted using a factorial within-subjects design. Patients (10 MS, 8 CVA) completed three tasks, each including a specific arm-movement along one of three directional axes (left-right, up-down and forward-backward).

**Results:**

The use of stereoscopy within a virtual training environment for neurorehabilitation of CVA and MS patients is most beneficial when the task itself requires movement in depth. Further, the 2.5D environment yields the highest efficiency and accuracy in terms of patients’ movements. These findings were, however, dependent on participants’ stereoscopic ability.

**Conclusion:**

Despite the performance benefits of stereoscopy, our findings illustrate the non-triviality of choices of using stereoscopy, and the type of graphic environment implemented. These choices should be made with the task and target group, and even the individual patient in mind.

## Background

In recent years many steps have been made towards the realization of rehabilitation using virtual environments. It has developed from an interesting technology with great potential for rehabilitation training to a realistic treatment option that is being deployed in clinical practice [1-4]. The aim of these systems is to offer a better and more efficient rehabilitation program that aids patients’ recovery of functions. The study presented in this paper is part of an international research project (INTERREG-IV-project “Rehabilitation Robotics II”) investigating the effects of arm training for MS (multiple sclerosis) and CVA (cerebrovascular accident, or stroke) patients when interacting in a 3-dimensional virtual (visual and haptic) environment (VE) using a robotic support system, with force sensing and force feedback. Both MS and CVA affect the central nervous system resulting in limited mobility of the lower and upper limbs, limitations in daily life function leading to dependency, and a reduced physical activity level, which can, in turn, lead to development of secondary diseases. When designing rehabilitation systems, well-informed choices need to be made about the graphical training environment (2.5D or 3D) and display techniques (monoscopic or stereoscopic image presentation) of the visual training environment. Although much is known about the impact of these variables on the perceived visual fidelity and performance characteristics, such data have typically been obtained using participants that have not suffered any neurological impairment. Little is known, however, about the influence of these variables on the efficiency, or accuracy with which different patient populations can interact with a VE.

The aim of the study presented in this paper is to assess how the graphic environment and stereo visualization influence movement efficiency and accuracy of MS and CVA patients’ movement (subjectively and objectively) in the VE, and to establish to what extent these factors can be beneficial or disruptive. The results will aid decisions when designing VEs for neurorehabilitation of MS and CVA patients.

### Neurorehabilitation and robotics

Virtual Environment (VE) technology and robotic systems offer opportunities to understand, measure, and treat a variety of clinical populations with central nervous system dysfunctions. The combination of a well-controlled, interactive, three-dimensional virtual environment with assistive robotic devices that can act both as sensor and actuator, provides clinicians with a useful toolset for the study and rehabilitation of perceptual, cognitive, and behavioral processes and functional (dis)abilities [5].

Within existing trials and training programs, a range of technologies is used that serve as the interface to the virtual world [c.f. 2]. Rehabilitation technology currently in use consists of electro-mechanical devices and robots like the HapticMaster [6], MIT-MANUS, or now the InMotion Shoulder-Elbow Robot [7], Brachio di fero [8], ARMEO Spring [9] and the BiManu-Track [10]. These devices have been designed to train the upper limbs and were all tested in at least one randomized clinical trial (RCT) [11].

Many of the interaction devices for upper limb rehabilitation allow interaction in three dimensions. As we have observed in informal pilot studies, some patients experience difficulty in navigating a 3D VE. From a clinician's point of view, however, 3D movements can be considered important for upper arm rehabilitation as they extend the range of movements available in training with a VE, and are closely related to movements made in activities of daily life (ADL). However, the feedback systems used, i.e. the displays, do not always reflect the three dimensional nature of the interface device in existing rehabilitation systems. Often, the visual display is a monoscopic screen, although there are examples of the use of stereoscopic display techniques in rehabilitation settings as well *e.g.*, [12-15]. What appears to be missing, is an understanding about when, and to what extent, stereoscopic visualization of virtual reality is beneficial or disruptive for patients using the system.

### Stereoscopy in the context of neurorehabilitation training

Stereoscopic techniques facilitate depth perception of a VE by supporting binocular vision. All stereoscopic display techniques rely on separately presenting an image to the left and right eye. These images contain a slight horizontal shift with respect to each other, thereby creating a point-to-point disparity variation across the two retinas, which the brain combines into a coherent perception of depth. Binocular vision offers advantages in certain tasks, especially in the comprehension of complex visual presentations and those requiring good hand—eye coordination. Stereoscopic visualization has been associated with improved judgments of three-dimensional spatial relationships *e.g.*, [16], more precise localization of objects [17], and faster performance in visually guided reaching tasks *e.g.*, [18]. These kinds of tasks are clearly relevant for rehabilitation training using VEs. In addition, stereoscopic displays have been associated with higher feelings of visual realism or presence [19]. Recent findings further indicate that stereoscopy also caters for a more natural mapping of people’s movement in the VE. For instance, gestures have been found to be more naturalistic and spatial in nature when using a stereo display compared to non-stereo conditions [20]. Grabbing and dragging, or grabbing and pushing, was more often observed in stereo than in non-stereo conditions. Gestures that are less spatial in nature, such as pinching in and out, were more frequently observed in non-stereo conditions.

Although stereoscopic display techniques offer clear potential benefits to patients by supporting a more detailed perception of depth, providing a more naturalistic mapping between 3D movements and 3D feedback, and allowing patients to be more engaged by the virtual environment, there are also potential drawbacks associated with stereoscopic displays. Both visual discomfort and visual fatigue have been reported when making use of stereoscopic displays [for an overview see: [21]. Negative effects associated with stereoscopic displays include people reporting a feeling of eyestrain, blurred vision, and focusing problems, all which can impact the usefulness of stereoscopic displays. In addition, immersive virtual environments have been reported to bring about a condition known as ‘simulation sickness’ or ‘cybersickness’. Although this condition seems to be rare in virtual rehabilitation [2], increased immersiveness of virtual environments (*e.g.*, by using stereoscopic presentation of the VE) may be more likely to bring about such negative effects.

While for healthy persons the downsides of stereoscopy mainly affects people's viewing comfort, for patient populations such effects could be detrimental to the main purpose of using a VE, which is rehabilitation. MS and CVA patient populations suffer from a variety of cognitive and visual disorders that make perception and spatial orientation in a virtual environment more difficult. Visual disorders are commonly observed for both MS and CVA patients, already taxing the visual system for these patients. Research estimates visual system disorders to be very common in MS patients with 80% presenting a visual impairment [22,23]. Ocular and visual deficits included here are diplopia, oscillopsia, blurred vision, loss of stereopsis, reading fatigue, reduction of contrast sensitivity, color perception, and visual acuity, and visual field defects. Furthermore, comparable visual and cognitive disorders are also prevalent in other neurologic disorders like stroke, with neglect of the environment being a common visuo-perceptual disorder. Between 30% and 85% of stroke patients will experience some type of visual dysfunction [24], and cognitive impairment affects 78% of stroke patients [25,26].

Given the likely constraints of MS and CVA patients due to visual and spatial cognition impairments, it is important to base decisions of using stereoscopy and rendering of the environment on *both* potential performance benefits (*e.g.*, efficiency and accuracy of movement) *and* potential negative effects like increased fatigue or discomfort. The aim of the current study was to establish the extent to which MS and CVA patients are able to benefit from 3D spatial representations in terms of both graphics content (2.5D vs 3D graphics) as well as information display (monoscopic *vs.* stereoscopic display modes). These results can then serve as input for the design of virtual environments for rehabilitation, in particular for implementation in the I-TRAVLE system (Individualized, Technology-supported and Robot-Assisted Virtual Learning Environments) that is developed within the Rehabilitation Robotics II project.

## Method

### Participants

Participants were recruited from the Rehabilitation and MS Centre Overpelt. The Ethical Committee of Hasselt University as well as the local Ethical Committee of the Rehabilitation and MS Centre in Overpelt (Belgium) approved the experimental protocol. After the participants gave their written informed consent they could participate in the study. Participants (N=18, 10 MS (5 male, 5 female), 8 CVA (4 female, 4 male; see Table 1), mean age was 57, with ages ranging between 36 and 79) were included who had a clinical diagnosis of MS or CVA and had no or only mild arm-hand dysfunction (Score Motricity Index ≥ 76) in at least one arm. They were excluded when an MS relapse took place in the last month before the test or when they had endured a CVA less than 6 months ago. Other exclusion criteria were: serious cognitive limits (Mini Mental state examination < 23), serious visual limits (Stereo blind on the Stereo Fly test, SFT = 0; low visual acuity on the E-chart test), neglect or apraxia. The study reported on in this paper was part of a larger study including additional conditions. Whereas most exclusion criteria were applied beforehand, in order to only invite eligible patients (N=20), stereo vision was only applied as an exclusion criteria for all conditions reported in this paper. This resulted in the exclusion of two patients from analysis presented in this paper based on their performance on the Stereo Fly test, indicating they were stereo blind.

**Table 1:** Clinical information regarding CVA patients

**Age**	**Gender**	**Type of CVA**	**Location of CVA**
66	Male	Ischemic stroke	Thalamic infarct left
74	Female	Ischemic stroke	-
79	Male	Ischemic stroke	subdural bleeding left
48	Female	Ischemic stroke	Right hemisphere – white matter
44	Male	Ischemic stroke	Left
60	Female	Ischemic stroke	Pure Sensory Lacunar stroke, Basal ganglia left and right + temporal lobe right + Semioval center right
55	Male	-	Left

note: Type and location of the CVA was unknown for 1 participant.

### Input device and setup

The input device used was a HapticMaster (MOOG), which provides 3 degrees of freedom (DOF) input and force feedback. A passive ADL gimbal (measurement of angles only, MOOG) was attached to the device’s endplate allowing participants to perform full arm movements, including rotational movements in the forearm (pro/supination). A thermoplastic brace attached to the MOOG ADL Gimbal connected participants to the HapticMaster. The gimbal allowed patients to interact with the device without the need to grip the haptic master with their hand, which is difficult for many persons in the target group. The HapticMaster was used to move a weightless virtual object (representing participants' current location in the VE) towards target locations within the VE, that is described in more detail elsewhere [27]. This VE was run on a computer with a 19” CRT screen as the visual output. The display was placed at a distance of 1,20 m in front of the subject (FOV=17,30 degrees). In the event of an emergency, both the participant and the therapist could switch off the system’s power, by operating a push button connected to a safety circuit.

### Design

An experiment was conducted manipulating Graphic Environment (2.5D *vs.* 3D) and Stereo Visualization (monoscopy *vs.* stereoscopy) of the VE for rehabilitation training of MS and CVA patients. A within-subjects design was used. The order of the conditions was randomized. After completing these conditions, participants were invited to complete two additional conditions in which the effectiveness of droplines and drop shadows was compared for the 3D mono conditions only [see: [28] for results].

### Experimental tasks

Three different skill components (lifting, transporting and reaching) that were identified as important for functional activities [29], were developed to serve as training tasks. Including these three tasks allowed a comparison of the influence of Graphic Environment and Stereo Visualization on task performance and experienced ease of localization for movement in the three primary movement directions of the tasks (horizontal, vertical and in depth). Lifting required the participants to move the virtual object to a target location above or below the initial cursor position. Transporting involved movement of the virtual object to a target location left or right from the initial cursor position. Reaching involved movement of the virtual object to a target location placed in front or behind the initial cursor position. All tasks were started with the HapticMaster moving the participant’s hand to the correct starting point (i.e. the initial cursor position).

### Manipulations

**Figure 1:**
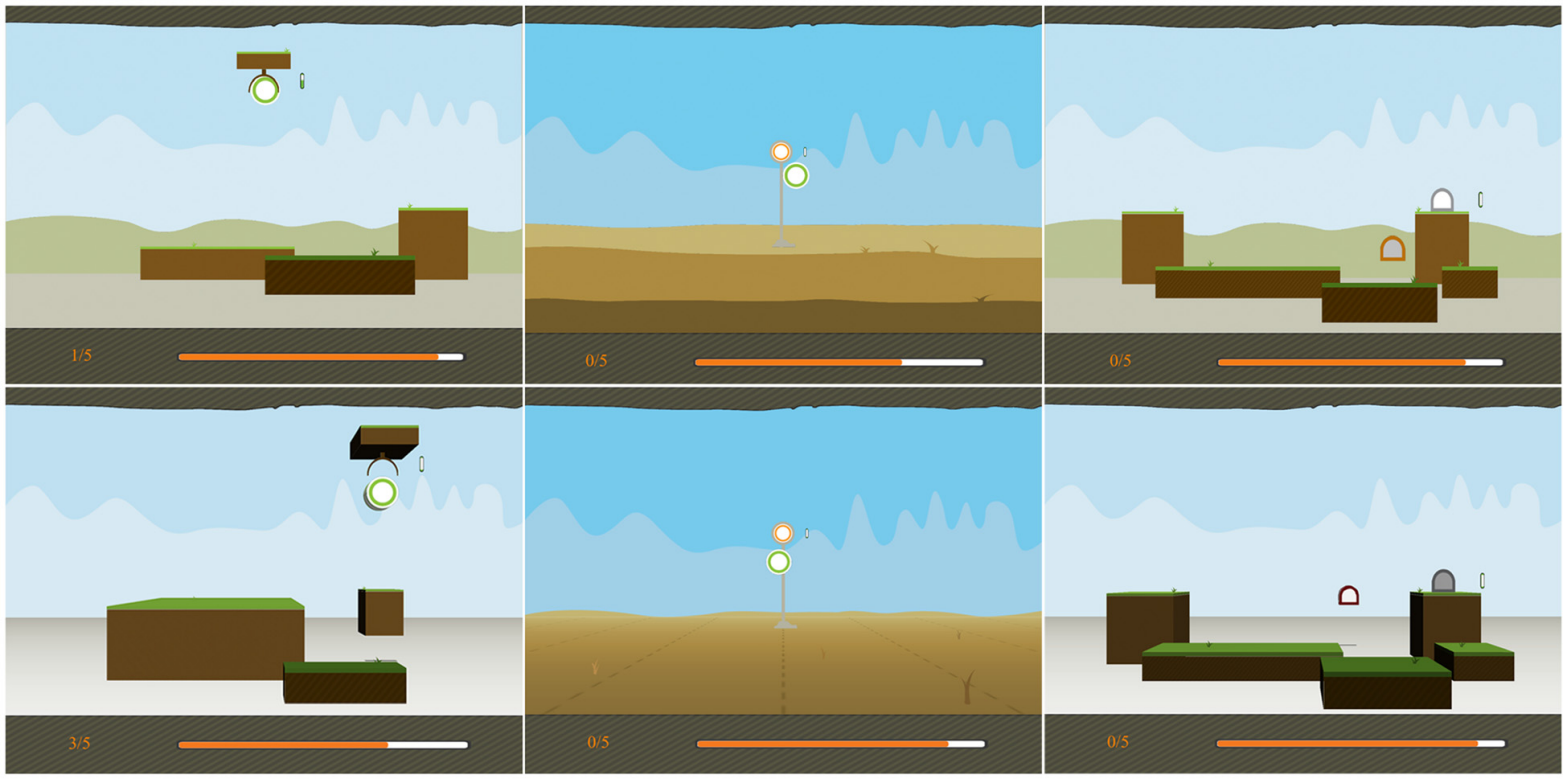
**Example of the three training tasks as used in the 3D conditions.** From left to right: lifting, reaching, and transporting. Top: 2.5D rendering, Bottom: 3D rendering.

The virtual environment consisted of a virtual world where participants could manipulate specific virtual objects. Two versions of the VE were built: a 2.5D and a 3D version (see Figure 1). The 2.5D version was built much like a puppet-theatre. It consisted of flat objects (depth of objects is zero) stacked behind each other. Although object movement was continuous, perspective cues were absent, rendering a discrete depth representation of the environment. The 3D version consisted of a VE where the objects were rendered using perspective cues, which yields a more continuous depth.

Stereo Visualization (monoscopic *vs.* stereoscopic) was manipulated by means of a time-multiplexed stereoscopic display, using shutter glasses. Shutter glasses facilitate a stereoscopic viewing experience by rapidly alternating vision through the left and right glasses in synchrony with images intended for the left or the right eye. The screen refresh rate was set at 120 Hz. The onscreen distance was set to 2 mm for the background, resulting in a disparity of 0.1 degrees (6 min/arc). This value is well within the comfort limit associated with stereoscopy, for normal populations [21]. Although for the monoscopic conditions there was no technical need to use the shutter glasses we had patients wear them nonetheless. This was done in order to make a fair and controlled comparison between monoscopic and stereoscopic conditions possible. Specifically, shutter glasses reduce the amount of light that reaches the eye, which makes both the room and stimuli darker. Should this reduction of light be exclusively associated with the stereoscopic viewing condition, this would introduce a systematic confound hampering our ability to attribute the results to the manipulation of stereo only. Further, only using the shutter glasses in the stereoscopic conditions could result in potential demand characteristics of the experiment, influencing people's reported experience in a systematic way. Between conditions, while filling in the questionnaire, the glasses were taken off.

### Measurements

Prior to the experiment, patients were tested for stereovision using the Stereo Fly Test (SFT, Stereo Optical Co. Inc.). During the experiment two sets of data for the dependent variables were collected: subjective self-report measurements and objective measurements of movement quality. Whereas the self-report measurements provided information about people’s subjective experience with the task (*e.g.*, visual comfort, ease of orientation), the objective measures provided information about people’s movement during the task (time on task, average speed of movement, and overshoot).

### Stereo-fly test

The stereo Fly Test is a test designed to measure people's stereovision. The score ranges from zero (stereo blind) to nine (perfect stereovision). A score of five or higher is generally accepted as indicating good stereovision, values below five indicate poor stereovision. A score of zero indicates stereo blindness. Two patients scoring a zero were excluded from the analyses. Seven participants (4 MS, and 3 CVA) fell into the poor stereovision condition (SFT<5), and 11 participants (6 MS, and 5 CVA) fell into the good stereovision condition (SFT 5 or higher). Scores of both patient groups (MS and CVA) were very similar and not significantly different (M_ms_=6.10, SD=2.85, M_CVA_=6.13, SD=3.76).

### Self-report measurement: ease of orientation

After completing the three tasks in each condition participants expressed their subjective ease of orientation. In total 6 questions were asked regarding the ease of orientation of both target and object in the virtual environment (see Table 2 for the questions used). Participants were asked to indicate to what extent they agreed with each of the statements. A four-point scale ranging from 0 (completely disagree) to 3 (completely agree) was used. The scores for the six items were averaged to form one scale indicating overall ease of orientation in the virtual environment. Reliabilities were calculated for each of the four conditions reported in this paper and were good to excellent (Cronbach’s alpha ranged between .83 and .95).

**Table 2:** Self-report measures of ease of orientation

**Questions**	
1	I found it easy to orient in the virtual environment
2	It was easy for me to locate the target
3	It was easy for me to reach the target
4	It was easy for me to locate the position of the moving object
5	It was clear to me in which direction I had to move
6	It was clear to me when I had to move the object in depth

### Self-report measurement: visual comfort

Participants reported their visual comfort after completion of each condition. Six questions were used measuring visual comfort: general uncomfortable feeling in the eyes, eye fatigue, the occurrence of headache, problems with focusing, experiences of blurred vision, and eyestrain. Each item was measured using a four-point scale (1=not, 2=light, 3=moderate, 4=strong). The question measuring general uncomfortable feeling was not analyzed, as it was found to be often misinterpreted by participants, who frequently reported uncomfortable feelings in different parts of their bodies, unrelated to visual (dis)comfort.

### Objective movement quality

In order to gather objective data about participants’ quality of movement during the experiment, data streams from the interaction device (HapticMaster) were logged with a sampling frequency of 120 Hz. The data included coordinates of the HapticMaster in 3 dimensions, and actual time. From this dataset a series of metrics quantifying movement quality for each of the tasks were computed: mean time for the completion of the trials (s), mean velocity for the trajectory performed during the trials (m/s), and mean overshoot for the trials (m). All measurements were calculated based on all the trials completed for each task separately (reach, lift and transport), per participant.

### Procedure

Participants were placed in front of the HapticMaster and were explained how to use the device. They were then asked to manipulate the HapticMaster in order to control a virtual object on a computer screen. It is important to note that the focus of the study in this stage is on Graphic Environment and Stereo Visualization of the training system rather than on the improvement of the upper limb movement of the patient. It was therefore decided that patients should execute all trials with their normal or less impaired limb. This allowed testing of the parameters under investigation with the relevant patient population, while minimizing influences of motor problems of the patients on task performance.

Once they could move the virtual object, the study started with a try-out exercise of five minutes, in which participants got used to the Haptic Master. After this short training the experiment started. Participants conducted all 4 conditions that made up the experimental design. In each condition patients performed three tasks (reaching, lifting, and transporting). Each task was performed for a maximum of 2 minutes, or a total of 5 repetitions of the task, whichever occurred first. After each condition, patients reported their experience with the training task by filling out a questionnaire. This questionnaire included a series of questions probing for potential negative effects of using the stereoscopic system (*e.g.* eye-strain, fatigue), and six questions probing for participant’s subjective ease of orientation in the virtual environment.

### Statistical analyses

Analyses consisted of a series of repeated measures ANOVA's (REMANOVA) on each of the dependent variables (visual comfort, subjective ease of orientation, and objective outcome measures). For each of the dependent variables a REMANOVA was conducted using Stereo Visualisation (monoscopy *vs.* sterescopy) and Graphic Environment (2.5D *vs.* 3D) as within subject variables. To control for the effect of stereovision stereovision (poor *vs.* good) was entered as an additional between subjects factor.

For visual comfort, 5 REMANOVA's were conducted, one for each variable. Subjective ease of orientation comprised one REMANOVA for the combined scale. For objective outcome measures REMANOVA's were conducted for each of the three dependent variables (time, speed, and overshoot) for the three tasks separately. For the objective outcome measures 9 REMANOVA's were conducted.

## Results

### Visual comfort

The analyses indicated that stereoscopy influenced reported experiences of fatigue and eyestrain. For fatigue, the interaction between Stereo Visualization and Stereovision was significant (*F*(1,16)=5.91, *p*=.027). More fatigue was reported with stereoscopic presentation (M= 1.36) as compared to monoscopic presentation for patients with weak stereovision. For patients with good stereovision there was a small inverse pattern (M_mono_=1.27, M_stereo_=1.14). For eyestrain a significant main effect of Stereo Visualization was found (*F*(1.16)=4.88, *p*=.042) with patients reporting more eyestrain in the stereoscopic(M=1.32) than in the monoscopic (M=1.20) condition.

### Subjective ease of orientation

Overall, participants experienced orientation in the tasks to be relatively easy (M=2.65, SD=0.58). The REMNOVA showed a main effect of Environment (*F*(1,16)=6.57, *p*=.021). Participants reported orientation to be easier in the 3D environment (M=2.75) as compared to the 2.5D environment (M=2.65) No other effects were significant. Means per condition: 2.5D mono = 2.63 (0.61), 2.5D stereo = 2.73 (0.48), 3D mono = 2.74 (0.42), and 3D stereo = 2.78 (0.46).

### Objective outcome measures

Objective outcome measures regarding patients’ movements in different conditions were collected for each of the three tasks (reach, transport and lift) separately. Three metrics (Average time, speed, and overshoot) were individually entered as the dependent variable. The results will be presented for each of the movement directions separately (see Table 3 for averages per condition).

**Table 3:** Objective outcome measures by condition per task

	**2.5D mono**	**2.5D stereo**	**3D mono**	**3D stereo**
		Reach		
Duration (s)	13.18 ±6.82	10.60 ±5.70	11.33 ±7.13	11.57 ±9.21
Speed (m/s)	0.10 ±0.039	0.13 ±0.047	0.13 ±0.055	0.13 ±0.048
Overshoot (m)	0.39 ±0.008	0.40 ±0.011	0.41 ±0.016	0.40 ±0.010
		Lift		
Duration (s)	16.10 ±8.76	15.79 ±8.37	18.94 ±14.32	16.13 ±5.93
Speed (m/s)	0.075 ±0.022	0.080 ±0.031	0.071 ±0.027	0.073 ±0.022
Overshoot (m)	0.034 ±0.025	0.030 ±0.019	0.029 ±0.026	0.027 ±0.018
		Transport		
Duration (s)	11.17 ±11.02	10.75 ±11.30	11.13 ±10.25	10.17 ±10.28
Speed (m/s)	0.12 ±0.033	0.13 ±0.042	0.12 ±0.040	0.13 ±0.038
Overshoot (m)	0.030 ±0.016	0.027 ±0.016	0.027 ±0.016	0.030 ±0.017

note: Values are means ± standard deviations.

### Reach

For average time there was a borderline significant main effect of Stereo Visualization, *F*(1,16)=3.70, *p*=.072, with a monoscopic presentation requiring more time (M=12.72s) than a stereoscopic one (M=11.55s). In addition, the interaction between Graphic Environment and Stereo Visualization, *F*(1,16)=4.70, *p*=.046 was significant, as was the interaction between Graphic Environment and Stereovision (*F*(1,16)=5.82, *p*=.028). More specifically, the interaction between Graphic Environment and StereoVisualization was such that especially in the 2.5D environment the stereoscopic condition required less time (M=10.80s) than the monoscopic condition (M=13.30s). There was little difference between the monoscopic and stereoscopic conditions in the 3D condition (M_3D_mono_=11.94, M_3d_stereo_=12.30). The interaction between Graphic Environment and Stereovision illustrated that for those with weak stereovision the 2.5D environment (M=13.33s) required less time than did the 3D environment (M=15.11s). For patients with good stereovision the opposite pattern was found, with movement in the 2.5D environment requiring more time (M=10.98) than in the 3D condition (M=9.13).

For average speed the main effect of Stereo Visualization was significant, *F*(1,16)=7.44, *p*=.015. In the monoscopic condition average speed was lower (M=0.11) than in the stereoscopic condition (M=.13). The interaction between Graphic Environment and Stereo Visualization was significant, *F*(1,16)=6.12, *p*=.025. This interaction indicated that the increase in speed for the stereoscopic condition was stronger in the 2.5D environment (M_2.5D___mono_=.10, M_2.5D_stereo_=0.13) than in de 3D environment (M_3D_mono_=.12, M_3D_stereo_=0.13). Additionally, there was a tendency towards significant interaction between Graphic Environment and Stereovision, *F*(1,16)=3.43, *p*=.083. This interaction indicated the average speed to be highest in the 3D environment, as compared to the 2.5D Environment, for those with good stereovision (M_2.5D_=.12, M_3D_=.14). For those with weak stereovision there was no difference in average speed between the two Environments (M_2.5D_=.12, M_3D_=.11).

For overshoot there was a significant main effect of Stereo Visualization, *F*(1,16)=5.56, *p*=.031, and a significant interaction between Graphic Environment and Stereo Visualization, *F*(1,16)-8.20, *p*=.011. These effects showed that overshoot was smaller in the stereoscopic conditions (M=.399) than in the monoscopic conditions (M=.404). This effect was, however, strongest in the 3D environment (M_mono_=.412, M_stereo_=.401). Stereo Visualization did not affect overshoot in the 2.5D environment (M_mono_=.396, M_stereo_=.397).

### Transport

For average time there was a significant main effect of Stereo Visualization, *F*(1,16)=5.13, *p*=.038 with less time required in the stereoscopic condition (M=11.12s) than in the monoscopic condition (M=11.96).

For average speed there was again a main effect of Stereo Visualization *F*(1,16), *p*=.033, with average speed being higher in the stereoscopic condition (M=.13) as compared to the monoscopic condition (M=.12).

For average overshoot the interaction between Graphic Environment and Stereovision was marginally significant, *F*(1,16)=4.04, *p*=.062. While for those scoring low on stereoscopic vision the 2.5D Environment tended to result in less overshoot (M=0.36) than the 3D condition (M=.042), for those scoring high on stereoscopic vision this appeared to be opposite (M_2.5D_=.024, M_3D_=.020).

### Lift

Regarding average time, the main effect of Graphic Environment, *F*(1,16)=6.19, *p*=.024, and the interaction between Graphic Environment and Stereovision, *F*(1,16)=5.30, *p*=.035) were significant. The results show that the lifting task required less time in the 2.5D environment (M=16.26) than in the 3D environment (M=18.26). The interaction illustrated that this effect was present only for those scoring low on stereoscopic vision (M_2.5D_=17.69, M_3D_=21.54). For average speed, like for average time, the main effect of Graphic Environment, *F*(1,16)=4.82, *p*=.043, and the interaction between Graphic Environment and Stereovision, *F*(1,16)=4.91, *p*=.042, were significant. The results show a pattern consistent with that found for average time. In the 3D environment (M=.078) speed was lower than in the 2.5D environment (M=.071). Again this was present only for those scoring low on stereoscopic vision (M_2.5D_=.081, M_3D_=.066). No significant effects for overshoot were found for the lifting task.

## Discussion

Using virtual environment (VE) technology in rehabilitation may facilitate the rehabilitation process by (i) allowing the systematic presentation of a varied set of rehabilitation exercises, tailored to the individual patient and his or her specific deficit, (ii) providing the therapist with additional quantitative assessment tools for diagnosis and progress monitoring, and (iii) providing the patient with purposeful and motivational environments which make it easier and more fun to adhere to a certain treatment protocol. For the rehabilitation of motor skills, a combination of VEs with haptic feedback and robotic technology is frequently used, in order to support and guide movements [1-4]. During the course of implementing VE rehabilitation program, clinicians are faced with many technology options and choices, that are not necessarily well understood in terms of their effects on a clinical population. One such option is whether or not to utilise stereo visualization as a way to present the rendered environment. Stereoscopic visualization has been associated with improved judgments of three-dimensional spatial relationships *e.g.*, [16], more precise localization of objects [17], faster performance in visually guided reaching tasks *e.g.*, [18], and a more natural mapping of people’s movement in the VE [20]. However, negative effects associated with stereoscopic displays include people reporting a feeling of eyestrain, blurred vision, focusing problems, all of which can impact the usefulness of stereoscopic displays [for an overview see: [21]. While using stereoscopic presentation of the VE could have beneficial effects for patients with neuropsychological deficits, it is also a potentially more vulnerable group which may already suffer from visual and spatial cognition deficits. It is unclear to what extent visualization and graphic rendering of the VE is beneficial or disruptive to patients’ navigation and experience of rehabilitation training. In the current study we investigated responses of patients with a clinical diagnosis of MS or CVA to manipulations of depth cues used within a rehabilitation VE. Specifically, we manipulated the dimensionality of the graphic environment (2.5D *vs.* 3D), and the use of stereoscopic presentation of the VE (monoscopic *vs.* stereoscopic).

Our findings illustrate that stereoscopic visualization of a VE can be beneficial over monoscopic visualization across patient groups. These benefits are, however, specific to the task that is being performed (reach, lift, and transport). Stereoscopic visualization was found to have positive effects on average speed, time spent on the tasks, and accuracy when the task itself included movement in depth (i.e., a reaching task). With a decrease in time, an increase in speed and an increase in accuracy it can be concluded that stereoscopy aided patients to reach the target more efficiently. When the task itself did not include movement in depth (lifting and transporting), these benefits were less marked. Although lacking functional use of the depth cues during lifting, there was evidence of increased speed and reduced time resulting from stereoscopy, but no effects were found regarding accuracy. This makes sense as the additional information provided by the stereoscopic visualization was to a lesser extent needed for the target localization during the vertical lifting task.

While these findings may lead to a conclusion that stereoscopic visualization is to be recommended in motor skills training for the patient groups under study, stereoscopic presentation did, however, result in more eyestrain in both MS and stroke patients. In addition, patients having weak stereovision reported higher fatigue. In this way, stereoscopy can also impede VR training. These are side effects that should be weighed carefully, as they potentially inhibit people's motivation to engage in training, leading to reduced training duration and reduced potential benefits for the recovery of arm-hand function. Since the potential motivational benefits of using VEs for rehabilitation training can be regarded as one of the key benefits from the patient’s point of view, negative effects on visual comfort may nullify such benefits. As such, current research regarding 3D visualizations and visual comfort are welcomed, while examinations with more recent systems are needed as stereoscopic systems continue to improve in image quality and visual comfort.

Besides influences of stereoscopy on time, speed and accuracy we also found evidence indicating that the graphic rendering environment (2.5D *vs.* 3D) influences these parameters. For the lifting task it was found that participants spent less time on the task and the average speed was higher in the 2.5D than in the comparative 3D environment. However, the stereoscopic ability varied considerably in each patient group, and clearly influenced the results. There was evidence that these benefits of the 2.5D environment over the 3D environment were most marked for those with weak stereoscopic vision. In contrast, for participants with good stereoscopic vision (SFT 5 or higher) the results suggest that the effects are opposite. For them, it appeared that the 3D environment reduced time on the task, increased average speed, and improved accuracy (reduced the average overshoot) as compared to the 2.5D condition. Although this finding has clear implications for the importance of tailoring the VE to the stereoscopic ability of individual patients, we do not have a readily available explanation for this finding. One potential explanation for this finding is that although visualization of a VE in 3D (as compared to 2.5D) improves the virtual environment in realism, by doing so part of the simplicity of the flat planes in the 2.5D environment is lost. Possibly, for those weak in stereoscopic ability, translating a richer VE (including a continuous 3D visualization for instance) might introduce an extra delay in interpreting object and target locations from the VE.

In developing virtual tools for rehabilitation training, we need to consider both patient characteristics (*e.g.*, level of stereoscopic ability), and define the functional parameters of the tasks (does the task require depth localization?). When depth cues like stereoscopic visualizations and 3D VE do not add value in terms of functionality (*e.g.*, depth cues are not needed *per se* when moving in the x-y plane), they may best be removed as we also found evidence that the use of stereoscopy increased visual fatigue for those participants with weak stereovision. If virtual learning environments are applied in patients with severe arm dysfunction, with the aim to aid patients in continued and intensive repetitive training, balancing person traits and task dependent functionality become important factors to consider.

A limitation to our current study is that participants used the stereoscopic mode of presentations for a limited duration only. The effect of stereoscopy on task performance and stamina in training when this system is used for prolonged periods of time is an important question, given that long training programs are needed to obtain functional training effects. It is a question that needs to be addressed in future research. Further, the effects of stereoscopy on task performance could increase when using larger disparities. In the current study we used a low disparity (0.1 degrees), which has been found to have relatively limited effects on performance measures [30]. A higher disparity would likely result in more pronounced effects on our objective performance measures, although it may also lead to a higher level of visual discomfort. Care should be taken that disparities should not exeed the 1 degree comfort limit [21], although it should be noted these limits have been established in relation to normal, unimpaired viewing populations, and not in relation to neuropsychological patient populations. Based both on our review of the literature and the findings of our study, we have reason to believe that patient populations suffering from functional deficits following brain damage may require a more conservative disparity limit so as to ensure visual comfort.

## Conclusion

Our findings illustrate that stereoscopy can have beneficial effects on efficiency and accuracy of movement in a virtual training tool for rehabilitation of MS and CVA patients. This is particularly true when depth is an integral part of the task at hand. When a task does not depend on movements in depth (i.e., the z-direction) but mostly involves movements between targets in the x-y plane, stereoscopy is unlikely to add value for the movement parameters (time, speeds, and accuracy) and type of tasks we employed in our study. The potential benefits of the use of stereoscopic displays in virtual rehabilitation need to be carefully weighed against the potential costs. In our study, stereoscopy increased reported eyestrain and visual fatigue, when compared to monoscopic modes of visualization. In sum, our findings demonstrate that technological parameters in virtual environments, such as graphic rendering and stereo visualization of the VE, need to be carefully tailored to the individual patient’s training needs and abilities, taking into account the particularities of each clinical population in terms of their visuo-spatial abilities. We are unlikely to find a one-size-fits-all solution for virtual rehabilitation environments, and it is important for both clinicians and VE designers to be aware of this.

## Competing interests

The authors declare that they have no competing interests.

## Authors’ contributions

WMVDH participated in the conception and design of the study, performed the statistical analyses, and drafted the manuscript. PF participated in the conception and design of the study, and helped draft the manuscript. IL carried out the study, and participated in the design of the study. KC participated in the conception and design of the study, and helped draft the manuscript. SN participated in the conception and design of the study, and carried out the study. LK carried out the study, and participated in the design of the study. WAIJ participated in the design of the study, and helped draft the manuscript. All authors read and approved the final manuscript.
